# Heparanase and Syndecan-4 Are Involved in Low Molecular Weight Fucoidan-Induced Angiogenesis

**DOI:** 10.3390/md13116588

**Published:** 2015-10-28

**Authors:** Oualid Haddad, Erwan Guyot, Nicolas Marinval, Fabien Chevalier, Loïc Maillard, Latifa Gadi, Christelle Laguillier-Morizot, Olivier Oudar, Angela Sutton, Nathalie Charnaux, Hanna Hlawaty

**Affiliations:** 1 Inserm U1148, Laboratory for Vascular Translational Science, UFR SMBH, Université Paris 13, Sorbonne Paris Cité, Groupe Biothérapies et Glycoconjugués, 93000 Bobigny, France; E-Mails: haddad.oualid@univ-paris13.fr (O.H.); erwan.guyot@jvr.aphp.fr (E.G.); nmarinval@yahoo.fr (N.M.); loic_maillard95@hotmail.fr (L.M.); latifa.gadi@yahoo.fr (L.G.); christelle.laguillier@jvr.aphp.fr (C.L.-M.); olivier.oudar@univ-paris13.fr (O.O.); angela.sutton@jvr.aphp.fr (A.S.); nathalie.charnaux@jvr.aphp.fr (N.C.); 2 Laboratoire de Biochimie, Hôpital Jean Verdier, Assistance Publique-Hôpitaux de Paris, 93140 Bondy, France; 3 ERL CNRS 9215, CRRET Laboratory, Université Paris Est Créteil, 94010 Créteil, France; E-Mail: fabien.che@gmail.com

**Keywords:** fucoidan, angiogenesis, human endothelial cell, glycosaminoglycan, syndecan, heparanase

## Abstract

Induction of angiogenesis is a potential treatment for chronic ischemia. Low molecular weight fucoidan (LMWF), the sulfated polysaccharide from brown seaweeds, has been shown to promote revascularization in a rat limb ischemia, increasing angiogenesis *in vivo*. We investigated the potential role of two heparan sulfate (HS) metabolism enzymes, exostosin-2 (EXT2) and heparanase (HPSE), and of two HS-membrane proteoglycans, syndecan-1 and -4 (SDC-1 and SDC-4), in LMWF induced angiogenesis. Our results showed that LMWF increases human vascular endothelial cell (HUVEC) migration and angiogenesis *in vitro*. We report that the expression and activity of the HS-degrading HPSE was increased after LMWF treatment. The phenotypic tests of LMWF-treated and *EXT2*- or *HPSE-*siRNA-transfected cells indicated that EXT2 or HPSE expression significantly affect the proangiogenic potential of LMWF. In addition, LMWF increased SDC-1, but decreased SDC-4 expressions. The effect of LMWF depends on SDC-4 expression. Silencing *EXT2* or *HPSE* leads to an increased expression of SDC-4, providing the evidence that EXT2 and HPSE regulate the SDC-4 expression. Altogether, these data indicate that EXT2, HPSE, and SDC-4 are involved in the proangiogenic effects of LMWF, suggesting that the HS metabolism changes linked to LMWF-induced angiogenesis offer the opportunity for new therapeutic strategies of ischemic diseases.

## 1. Introduction

Heparan sulfate proteoglycans (HSPG) are integral components of the cell surface and extracellular matrix (ECM) of animal cells. These complex molecules consist of sulfated carbohydrate chains of glycosaminoglycans (GAGs) covalently bound to a protein core. The GAGs are composed of disaccharide units composed of galactose or glucuronic/iduronic acid and *N*-acetyl-glucosamine/-galactosamine. Heparan sulfate (HS) chain compositions are cell-specific and can evolve during the development or tissue regeneration. All HS chains are synthesized *de novo*, and it is still unclear what determines their structures. The enzymes involved in the biosynthesis of a carbohydrate chain have been identified. Two main categories have been described: the enzymes responsible for chain polymerization, mainly exostosin-1 and -2 (EXT1, EXT2), and the enzymes responsible for chain modifications (*N*-deacetylase/*N*-sulfotransferases (NDST), 2-*O*-sulfotransferases (2OST), 3-*O*-sulfotransferases (3OST), 6-*O*-sulfotransferases (6OST), and heparanase (HPSE). Syndecans (SDCs) are heparan-sulfate containing transmembrane proteoglycans [1]. They bind various components of the ECM and are important regulators of cell-cell and cell-ECM interactions. Syndecans are involved in cell migration and angiogenesis [2,3]. Syndecan-4 (SDC-4) is a component of the focal contacts and its activation is associated with cell cytoskeletal rearrangement leading to cell migration [4]. Syndecan-1 (SDC-1) modulates β-integrin- and interleukin-6-dependent breast cancer cell adhesion and migration, and its overexpression in human fibrosarcoma cells leads to increased proliferation and migratory ability [5,6]. Low molecular weight fucoidan (LMWF), a sulfated polysaccharide from brown seaweeds that mimics some biological activities of heparin, has been shown to promote revascularization in a rat critical hindlimb ischemia [7]. It increases human vascular endothelial growth factor (VEGF_165_)-induced endothelial cell migration by enhancing VEGF_165_ binding to VEGFR-2 and neuropilin 1 (NRP1) [8]. Furthermore, fucoidan induces the adoption by endothelial colony-forming cells (ECFC) of an angiogenic phenotype *in vitro* and greatly increases ECFC-mediated angiogenesis *in vivo* [9]. We have previously demonstrated that LMWF, injected in rats, prevented intimal hyperplasia in the thoracic aorta by increasing human vascular endothelial cell (HUVEC) migration, but decreasing vascular smooth muscle cell migration through the modulation of matrix metalloproteinase-2 (MMP-2) expression [10]. Nevertheless, the effects of fucoidan on angiogenesis are somehow controversial. Indeed, depending on the seaweed origin, the sulfatation level, or the size, fucoidan may have antiangiogenic effects. Soeda *et al.* reported that oversulfated fucoidan (100 kDa) from *Fucus vesiculosus* significantly inhibited the fibroblast growth factor-2 (FGF-2) induced HUVEC migration and tube formation by increasing the release of plasminogen activator inhibitor-1 (PAI-1) [11]. In contrast, Kim *et al.* reported that fucoidan acts synergistically with FGF-2 in promoting HUVEC proliferation and agiogenesis by AKT and MMP-2 signalling via activation of the p38 and JNK signalling pathways [12]. In this study, we hypothesized that LMWF (8 kDa) from *Fucus vesiculosus* can also modify the amount and the distribution of heparan sulfate (HS) chains exposed at the endothelial cell surface and of two major heparan sulfate membrane proteoglycans, SDC-1 and SDC-4, causing modifications of cell properties related to proangiogenic abilities.

## 2. Results and Discussion

### 2.1. Effects of LMWF on Endothelial Cell Abilities (Migration and 2D-Angiogenesis)

**Figure 1:**
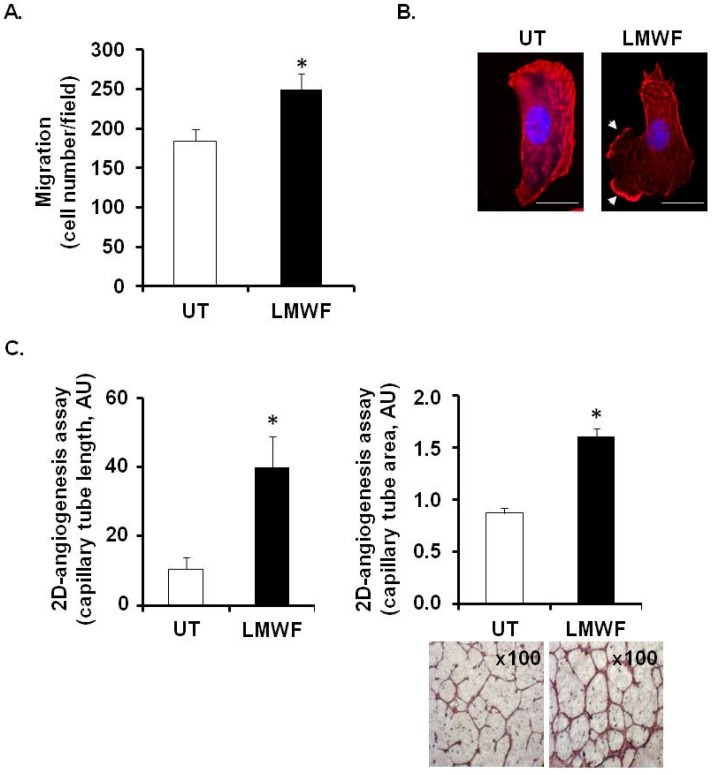
Effects of Low molecular weight fucoidan (LMWF) on endothelial cell abilities: migration and 2D-angiogenesis. Human vascular endothelial cells (HUVEC) were incubated with 10 µg/mL LMWF for 24 h and the migration (**A**), the lamellipodia formation (**B**) and the capillary tube formation (length and area) (**C**) were determined. (**A**) Migration chamber assay. HUVECs incubated with or without 10 μg/mL LMWF, were allowed to migrate through the porous fibronectin-coated membrane. They were stained with Mayer’s hemalum and counted. The results are expressed as cell number per field; (**B**) Lamellipodia formation. LMWF induced the formation of lamellipodia and ruffles (white arrows indicate lamellipodia/ruffle formation, DAPI-nucleus (blue), Phalloidin-F-actin (red)). Bar = 10 µm; (**C**) Capillary tube formation (2D-angiogenesis assay) on Matrigel. Left and right panels show the length (**left**) and area (**right**) of endothelial capillaries formed by HUVECs treated with or without 10 µg/mL LMWF. Lower right panel shows a representative image of capillary network, as photographed with phase contrast microscopy (magnification ×100). * *p* < 0.05 *versus* control untreated (UT) cells. A.U.: arbitrary unit.

LMWF at 10 μg/mL, but not high molecular weight fucoidan (HMWF) (600 kDa) increased HUVEC migration through fibronectin-coated 8 µm-porous membranes by 36% ± 8% (Figure 1A and data not shown). Confocal microscopy confirmed that LMWF induced the formation of lamellipodia and ruffles, which characterize a migration phenotype and reorganized actin cytoskeleton (Figure 1B). Using a 2D-angiogenesis assay, we demonstrated that LMWF induced the formation of capillary tubes in Matrigel by increasing their length by 4 fold and their area up to 84% ± 8%, as compared to untreated (UT) control cells (Figure 1C).

### 2.2. LMWF and Level of Glycosaminoglycan Chains Expressed in HUVECs

**Figure 2:**
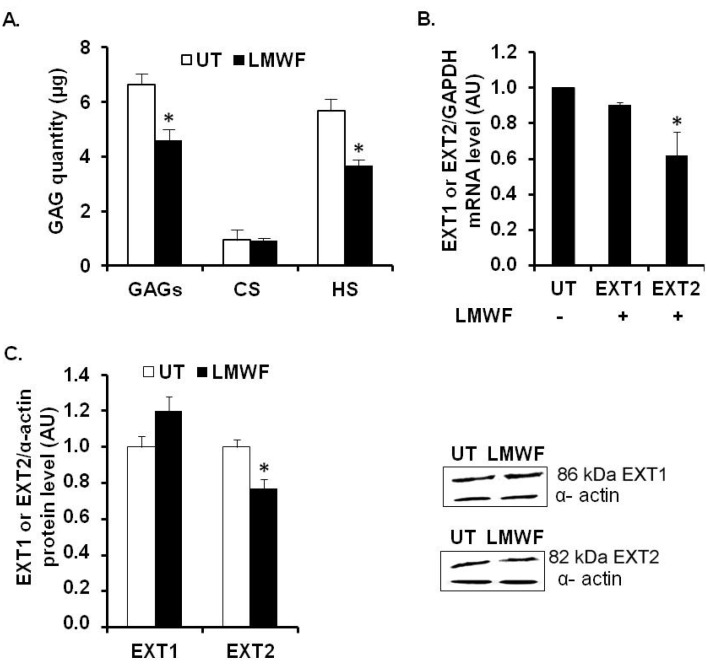
LMWF and glycosaminoglycan chain level in HUVECs. (**A**) Glycosaminoglycan quantification. HUVECs were incubated with 10 µg/mL LMWF for 24 h and the amount of total GAGs, CS and HS chains were determined in the supernatant according to a dimethyl-methylene blue (DMMB) assay; (**B**) Exostosin-1 and -2 (EXT1 or EXT2) mRNA levels were determined by real-time RT-PCR in cells treated with or without 10 µg/mL LMWF. (**C**) EXT1 or EXT2 protein levels were determined by western blot in cells treated with or without 10 µg/mL LMWF. Right panel shows a representative image of the western blot assay. * *p* < 0.05 *versus* control untreated (UT) cells. A.U.: arbitrary unit.

We first investigated whether LMWF could modify the GAG chain level expressed by HUVECs. For that purpose, the level of total GAGs, HS chains, and chondroitin sulfate (CS) chains were determined by DMMB assays in the lysate of endothelial cells after 24 h of 10 μg/mL LMWF treatment and compared to UT control cells. There was no significant difference in the level of GAGs, HS, and CS after LMWF incubation (Figure S1). Following LMWF treatment, the amount of total GAGs in the conditioned medium of LMWF-treated cells decreased by 28% ± 8% at 24 h, as compared to untreated control cells (*p* < 0.05, *n* = 3, Figure 2A). Further analysis revealed that HS amounts decreased by 25% ± 5% in the conditioned medium of the LMWF-treated cells, whereas there was no variation of CS chain amount (Figure 2A). These data suggests that LMWF may modify the HS and HSPG turnover (HS synthesis or cleavage and HSPG shedding).

### 2.3. LMWF and Heparan Sulfate Biosynthesis and Degradation Enzymes in HUVECs

We have first studied the effects of LMWF on enzymes involved in HS biosynthesis (EXT1, EXT2) or degradation (heparanase). These glycosyltransferases EXT1 and EXT2 are responsible for the elongation of HS by catalyzing the addition of alternating β-d-glucuronate (GlcA) and α-d-*N*-acetylglucosamine (GlcNAc) units to the tetrasaccharide linker of GAGs. We assessed the glycosaminoglycan polymerization (*EXT1* and *EXT2*) mRNA levels by quantitative RT-PCR. The mRNA expression level of *EXT2* in LMWF-treated cells was decreased by 36% ± 13%, as compared to untreated cells at 24 h (*p* < 0.05), whereas the level of mRNA encoding for *EXT1* was unaffected (Figure 2B). The EXT1 and EXT2 protein levels were measured by western blot analysis in HUVEC lysates. A slightly decreased EXT2 level by 23% ± 5% was observed in the LMWF-treated cells (*p* < 0.05). No significant difference was observed for EXT1 (Figure 2C).

**Figure 3:**
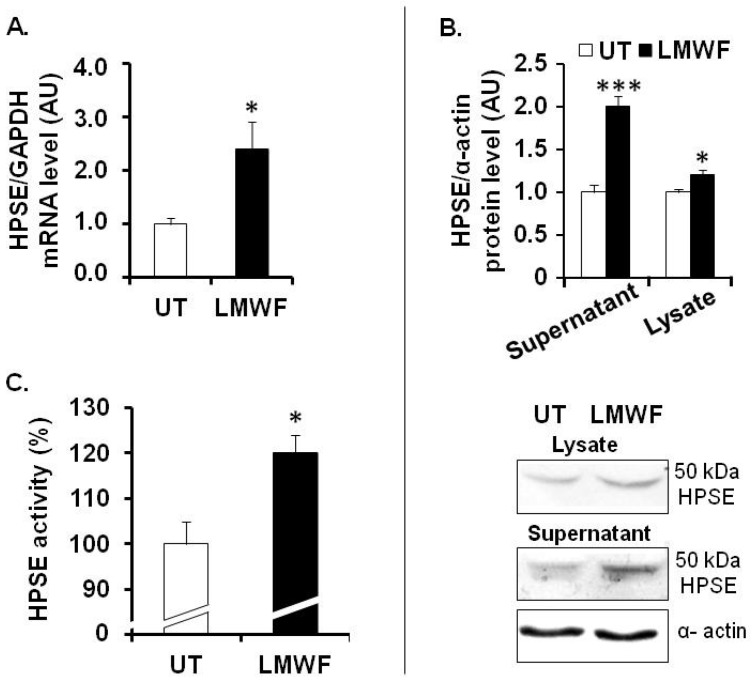
LMWF and heparanase in HUVECs. (**A**) *HPSE* mRNA levels were determined by real-time RT-PCR in cells treated with or without 10 µg/mL LMWF; (**B**) HPSE protein levels were determined by western blot in the supernatant or in the lysate of cells treated with or without 10 µg/mL LMWF. Lower panel shows a representative image of the western blot assay. (**C**) Heparanase activity was checked in the lysate of LMWF-treated cells. HPSE activity in untreated cells was arbitrary set to 100%. * *p* < 0.05, *** *p* < 0.0005, LMWF-treated cells *versus* LMWF-untreated cells (UT). A.U.: arbitrary unit.

**Figure 4:**
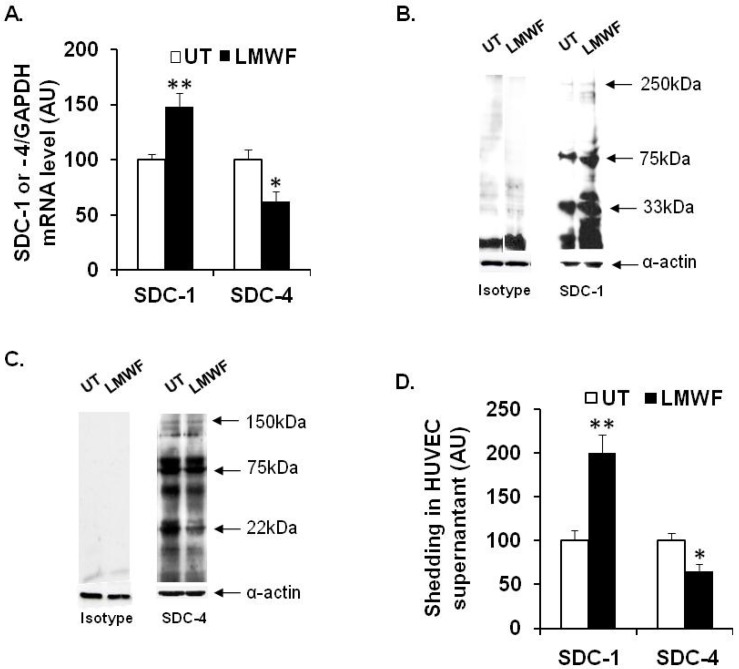
Effects of LMWF on the SDC expression in HUVECs. *SDC-1* and *SDC-4* mRNA or protein levels in endothelial cells treated or not with 10 µg/mL LMWF were analyzed respectively by real time RT-PCR (**A**) or western blot (**B**,**C**). SDC-1 and SDC-4 ectodomains in the supernatant of cells treated with or without 10 µg/mL LMWF were analyzed by dot blot (**D**). * p < 0.05, ** p < 0.005, significantly different to LMWF-untreated cells (UT). A.U.: arbitrary unit.

The HS-degrading enzyme heparanase (HPSE) is an endo-β-d-glucuronidase, which plays an important role in remodeling of the basement membrane and extracellular matrix during process of inflammation [13,14,15]. HPSE is synthesized as an inactive 65 kDa pro-form enzyme (pro-HPSE), can be transformed into active heterodimer consisting of 50 and 8 kDa subunits (active HPSE), and cleaves HS chains attached to proteoglycans, such as syndecans and perlecan [15,16]*. HPSE* mRNA expression, as assessed by quantitative RT-PCR, was increased by 2.4 fold in LMWF-treated cells, as compared to untreated control cells (Figure 3A). Active HPSE form (50 kDa) expression, as assessed by western blot, was significantly increased up to 2 fold in the supernatant or by 20% ± 5% in the lysate of the LMWF-treated cells (Figure 4B). HPSE activity was slightly but significantly increased up to 20% ± 3% in the lysate of LMWF-treated cells (Figure 3C), whereas it was not detected in the respective conditioned medium.

### 2.4. Effects of LMWF on the Syndecan Expression

We therefore focused on the effect of LMWF on the expression of the heparan sulfate transmembrane proteoglycans belonging to the syndecan family, SDC-1 and SDC-4.

In HUVECs, we demonstrated that LMWF increased the level of mRNA encoding for SDC-1 by 48% ± 12% (*p* < 0.005), whereas it decreased that of SDC-4 by 38% ± 9% (*p* < 0.05) (Figure 4A). We then analyzed SDC-1 and SDC-4 levels by western blot. The SDCs contains the core protein (ectodomain, transmembrane, and cytoplasmic domains) and the GAG, which are attached in the ectodomain part of SDCs. As shown previously, it is well known that the western blot expression pattern is heterogeneous (many bands from 20 kDa to 250 kDa) and show many forms of SDC-1 and SDC-4 in the cell lysate [17]. Our results of SDC protein expression showed the presence of 3 different forms for SDC-1 and SDC-4 (Figure 4B,C). Upon LMWF treatment, protein level was significantly increased by 22% ± 2% (33 kDa), 13% ± 5% (75 kDa) and 18% ± 4% (250 kDa) for SDC-1 or decreased by 28% ± 5% (22 kDa), 41% ± 9% (75 kDa), and 48% ± 5% (150 kDa) for SDC-4, respectively (Figure 4B,C). As assessed by dot blot, the shedded ectodomain level of SDC-1 in the supernatant of LMWF-treated cells was increased by 2 fold, as compared to untreated control cells, whereas that of SDC-4 was decreased by 35% ± 8% (*p* < 0.05) (Figure 4D).

Taken together, LMWF modulate SDC-1 and SDC-4 gene expression and ectodomain shedding in HUVEC *in vitro* culture.

*In vivo*, the influence of LMWF on SDCs expression was assessed in a Sprague Dawley Rat model of intimal hyperplasia. We have already used this model to show the pro-angiogenic effect of LMWF treatment [10]. In addition, SDC-1 and SDC-4 have been shown to play an important role in the pathological response of a balloon-injured wall artery [18]. Briefly, rats were subjected to balloon injury into the thoracic artery to create local destruction of endothelial layer leading to inflammation and intimal hyperplasia development. Two weeks after LMWF-intramuscular injection the level of SDC-1 and SDC-4 in the balloon-injured artery was analyzed.

**Figure 5:**
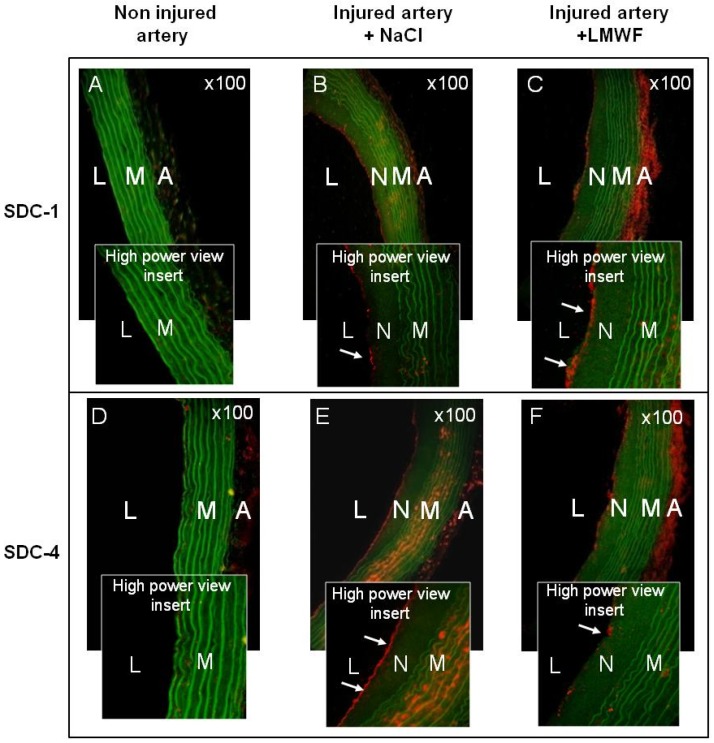
Effects of LMWF on the SDC distribution in rat balloon injured artery. SDC-1 and SDC-4 expressions were assessed using immunohistochemistry in rat model of intimal hyperplasia. (**A**) SDC-1 or (**D**) SDC-4 expressions in non injured arteries; (**B**) SDC-1 or (**E**) SDC-4 expressions in injured arteries treated with NaCl; (**C**) SDC-1 or (**F**) SDC-4 expressions in injured arteries treated with LMWF. White arrows indicate SDC expressions in the neointima layer in high power view inserts (red). Green: autofluorescence of the elastic fibers of the lamina. Magnification ×100, L: lumen, N: neointima, M: media, A: adventitia.

Our results demonstrated that the expression of SDC-1 and SDC-4 was very low in healthy arteries in the media (M) and adventitia (A) layers, whereas it increased in the neointima (N), media and adventitia layers in balloon-injured, and NaCl-treated arteries (vehicle), leading to development of intimal hyperplasia (Figure 5A–E). The LMWF treatment of injured artery increased SDC-1 expression in the neointima and in the adventitia layers, as compared to vehicle (Figure 5C). Furthermore, upon LMWF treatment, the SDC-4 expression was decreased in the neointima and media, but largely increased in the adventitia layer, as compared to vehicle (Figure 5F). These *in vivo* results were in concordance with our previously described observation obtained in the HUVECs *in vitro* culture [10].

All these results suggest that LMWF has an important influence on the proteoglycan distribution in the endothelial cells and can increase SDC-1 and decrease SDC-4 expression *in vitro* and *in vivo*.

### 2.5. Assessment of EXT-, HPSE- or SDC Involvement in Biological Effects of LMWF

To assess the potential role of EXT1/EXT2, HPSE or SDC-1/SDC-4 in the biological effects induced by LMWF, specific siRNAs were carried out.

**Figure 6:**
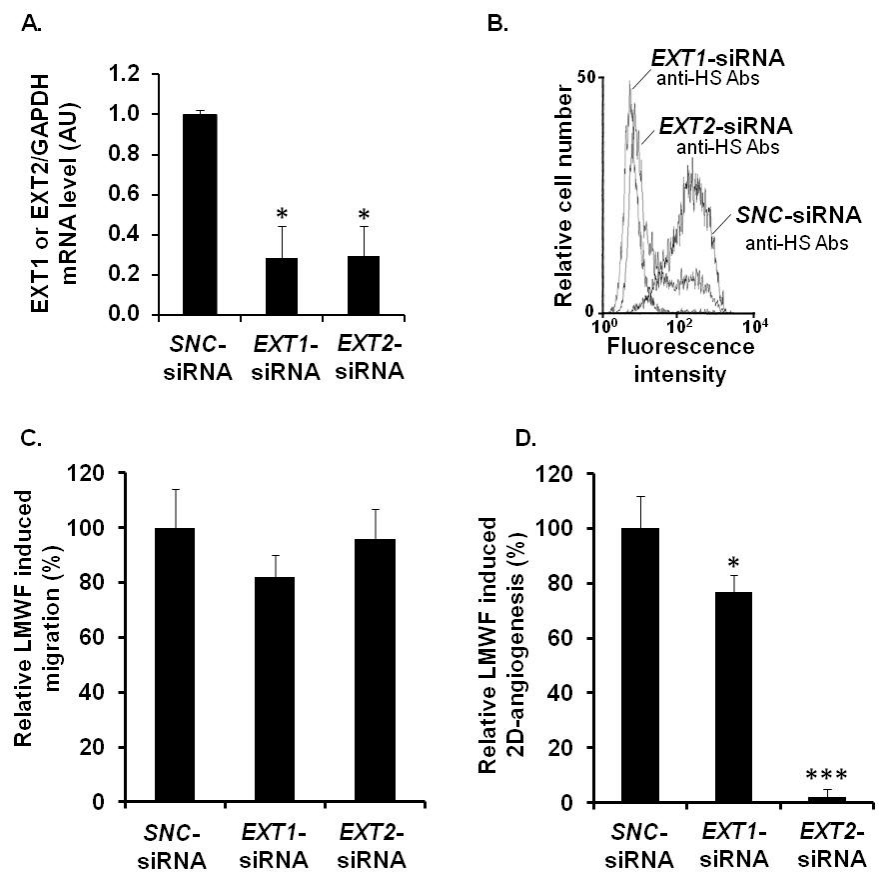
Assessment of EXT involvement in biological effects of LMWF in HUVECs. HUVECs were transfected with *EXT1*-siRNA or *EXT2*-siRNA or with *SNC*-siRNA control. (**A**) *EXT1* or *EXT2* mRNA levels were determined in *EXT1*-siRNA- or *EXT2*-siRNA- or *SNC*-siRNA-transfected control cell by real-time RT PCR. *EXT1*- or *EXT2* mRNA level normalized to *GAPDH* mRNA level in *SNC*-siRNA-transfected control cells was arbitrarily set to 1; (**B**) The binding of 10E4 anti-HS antibodies to *EXT2*- or *EXT1*-siRNA transfected cells was compared to that of *SNC*-siRNA-transfected cells; (**C**) Migration was assayed in cells treated with or without 10 µg/mL LMWF; (**D**) 2D-angiogenesis was assayed in cells treated with or without 10 µg/mL LMWF. The difference in the capillary network length between LMWF-treated and untreated cells in each RNA interference condition (*EXT1 or EXT2* silencing) was compared to that in *SNC*-siRNA-transfected cells. Control LMWF induction was arbitrary set at 100% for *SNC*-siRNA-transfected cells. * p < 0.05, *** p < 0.0005 *versus SNC*-siRNA-transfected control cells. A.U.: arbitrary unit.

Quantitative RT-PCR showed that the expression of the mRNAs and proteins of EXT2 in *EXT2-*siRNA- and EXT1 in *EXT1*-siRNA-transfected cells was reduced up to 72% ± 16% and 71% ± 15%, respectively, as compared to the *SNC*-siRNA-transfected control cells (Figure 6A). The flow cytometry assay showed that the binding of 10E4 anti-HS antibodies to *EXT2-*siRNA- or *EXT1-*siRNA-transfected cells was respectively reduced by 75% ± 8% or by 80% ± 9%, as compared to *SNC*-siRNA-transfected control cells (Figure 6B).

There were no significant difference in cell migration after LMWF treatment in *EXT1*-siRNA- or *EXT2-*siRNA-transfected cells, as compared to *SNC*-siRNA-transfected cells (Figure 6C). In contrast, the LMWF induction of 2D-angiogenesis was abolished in LMWF-treated *EXT2-*siRNA-transfected cells by 98% ± 5%, or decreased in LMWF-treated *EXT1-*siRNA*-*transfected cells by 33% ± 5% (*p <* 0.05), as compared to *SNC*-siRNA-transfected control cells (Figure 6D). These latter data suggest that EXT2 and, to a lesser extent EXT1, affect the pro-angiogenic effect of LMWF.

**Figure 7:**
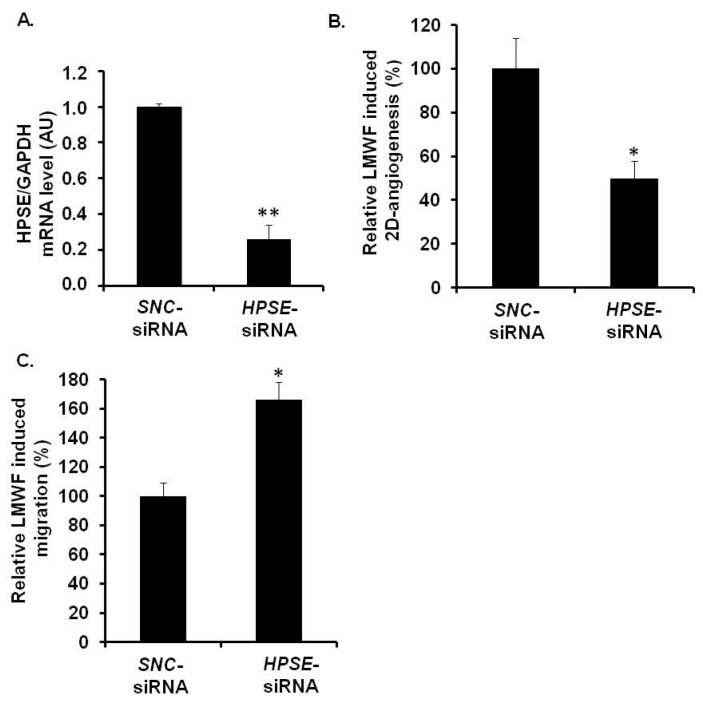
Assessment of HPSE involvement in biological effects of LMWF in HUVECs. HUVECs were transfected with *HPSE*-siRNA or *SNC*-siRNA control. (**A**) *HPSE* mRNA levels were determined in *HPSE*-siRNA- or *SNC*-siRNA-transfected cells by real-time RT-PCR. *HPSE* mRNA level normalized to *GAPDH* mRNA level in *SNC*-siRNA-transfected control cells was arbitrary set to 1; (**B**) 2D-angiogenesis was assayed in cells treated with or without 10 µg/mL LMWF. The difference in the capillary network length between LMWF-treated and untreated cells in *HPSE* RNA interference condition was compared to that in *SNC*-siRNA-transfected cells; (**C**) Migration was assayed in cells treated with or without 10 µg/mL LMWF. Control LMWF induction was arbitrary set at 100% for *SNC*-siRNA-transfected cells. * p < 0.05, ** p < 0.005 *versus SNC*-siRNA-transfected control cells. A.U.: arbitrary unit.

*HPSE-*siRNA-transfected cells were used for 2D-angiogenesis or migration assays. Quantitative RT-PCR showed that the expression of the mRNAs encoding for heparanase in *HPSE-*siRNA-transfected cells was reduced up to 74% ± 8%, as compared to the *SNC*-siRNA-transfected control cells (Figure 7A). Under basal conditions (in the absence of LMWF), *HPSE-*siRNA transfection decreased endothelial cell migration by 37% ± 5% (*p* < 0.05), but had no effect on 2D-angiogenesis (Figure S2A,B). However, upon LMWF stimulation and *HPSE*-siRNA transfection, the ability of HUVECs to form capillary network in Matrigel 2D-angiogenesis assay was altered. The capillary network length induced by LMWF treatment was largely decreased by 51% ± 11% in *HPSE*-siRNA-transfected cells, as compared to *SNC*-siRNA-transfected control cells (Figure 7B). In contrast, the cell migration induced by LMWF was significantly increased by 56% ± 9% (*p* < 0.05) in *HPSE*-siRNA-transfected, as compared to *SNC*-siRNA-transfected cells (Figure 7C).

LMWF biological effects have been checked in *SDC-1*-siRNA- or *SDC-4*-siRNA-transfected cells or in cells treated with specific anti-SDC-1 or anti-SDC-4 antibodies. As described [19,20], quantitative RT-PCR showed that the expression of the mRNAs encoding for SDC-1 in *SDC1-*siRNA- or SDC-4 in *SDC4*-siRNA-transfected cells was reduced up to 69% ± 14% and 73% ± 17% respectively, as compared to *SNC*- siRNA-transfected control cells.

**Figure 8:**
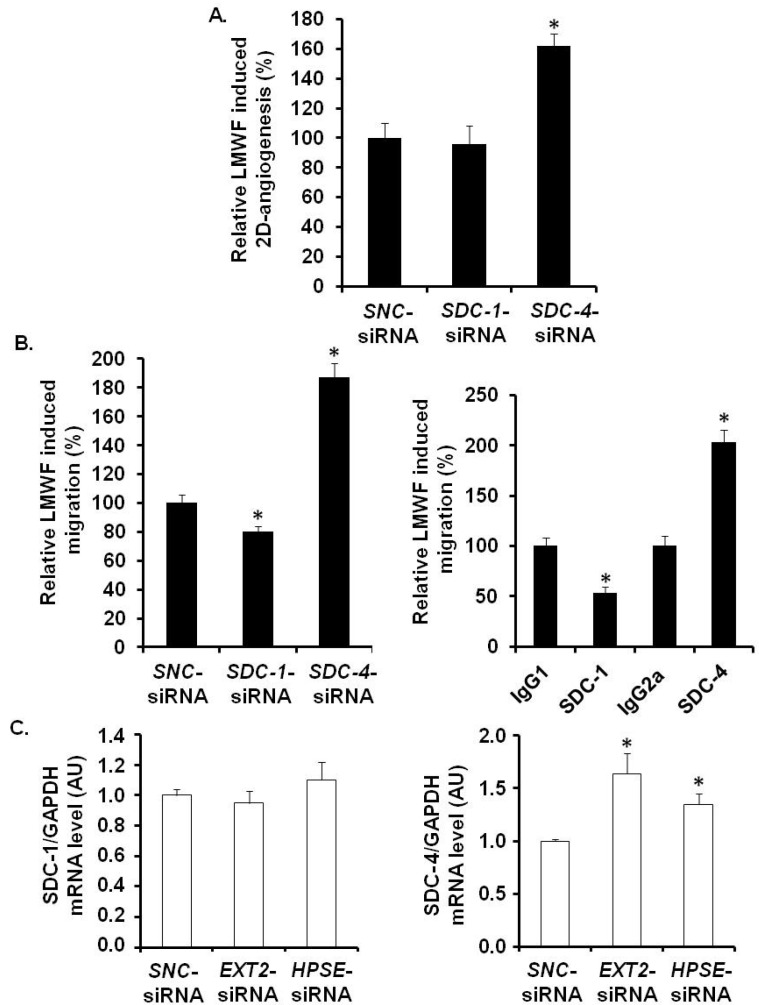
Assessment of SDC involvement in biological effects of LMWF in HUVECs. 2D-angiogenesis (**A**) and migration (**B**) assays were performed in *SDC-1*-siRNA- or *SDC-4*-siRNA- or *SNC*-siRNA-transfected control cells (**B**, **left panel**), or in cells treated with specific anti-SDC-1 or anti-SDC-4 antibodies (**B**, **right panel**). Control LMWF induction was arbitrary set at 100% for *SNC-*siRNA-transfected cells or isotypes. (**C**) *SDC-1* and *SDC-4* mRNA expression was analyzed in *EXT2*- or *HPSE*-siRNA-transfected cells. * p < 0.05 *versus SNC*-siRNA-transfected control cells or isotypes. A.U.: arbitrary unit.

Under basal conditions, the 2D-angiogenenis assays showed a significant decrease in cell capillary network length by 23% ± 4% in *SDC-1*- and by 54% ± 7% in *SDC-4*-siRNA-transfected cells, as compared to *SNC*-siRNA-transfected control cells (Figure S3A). Upon LMWF cell treatment, the LMWF-induction of 2D-angiogenenis was unchanged in *SDC-1*-siRNA-transfected cells, whereas it was significantly increased in *SDC-4*-siRNA*-*transfected cells by 62% ± 5% (*p <* 0.005), as compared to *SNC*-siRNA-transfected control cells (Figure 8A).

Under basal conditions, endothelial cell migration assayed in modified Boyden chambers was decreased by 43% ± 5% and by 40% ± 8% in *SDC-1*-siRNA-transfected and anti-SDC-1 antibody-incubated cells, respectively (Figure S3B). LMWF induction of endothelial cell migration was decreased in *SDC-1*-siRNA-transfected cells by 20% ± 5% and in anti-SDC-1 antibody-incubated cells by 47% ± 3%, as compared to respective control cells (Figure 8B). These data were confirmed by a wound healing assay (Figure S3C). Regarding RNA silencing experiments, these data suggest that SDC-1 does not play a crucial role in LMWF-induced effects.

Basal endothelial cell migration was decreased by 68% ± 5% and by 67% ± 9% in *SDC-4*-siRNA-transfected and anti-SDC-4 antibody-incubated cells, respectively (Figure S3B). However, LMWF-induction of endothelial cell migration was largely increased by 87% ± 5% in *SDC-4*-siRNA-transfected cells or by 2 fold in anti-SDC-4-antibody incubated cells, as compared to respective control cells (Figure 8B). These data were confirmed by a wound healing assay (Figure S3C). These results demonstrated that SDC-4 expression limits the LMWF effect on the cells.

Altogether, we have demonstrated that on the one hand EXT2 (and EXT1 to a lesser extent) and HPSE expression, and on the other hand SDC-4, play critical roles in LMWF pro-angiogenic effects. We have then addressed the question whether silencing of endothelial HPSE or EXT2 could affect SDC-4 level. In *HPSE*- or *EXT2*-siRNA-transfected cells, *SDC-4* mRNA level was up-regulated respectively by 64% ± 19% or 35% ± 10%, as compared to *SNC*-siRNA-transfected control cells (Figure 8C). In addition, there was no effect on *SDC-1* mRNA level in *HPSE*- or *EXT2* silenced cells (Figure 8C).

### 2.6. Discussion

Fucoidan exhibits various biological effects, among them anti-inflammatory, low anti-coagulant and anti-thrombotic activities. We have previously shown the therapeutic potential of low molecular weight fucoidan (LMWF) in reduction of in-stent restenosis in a rabbit model, vascular tissue repair [21], and in critical hind limb ischemia in a rat model [7].

In this study, we hypothesized that LMWF could modify the amount and the distribution of heparan sulfate chains expressed in endothelial cells and of syndecan-1 (SDC-1) and syndecan-4 (SDC-4), two major heparan sulfate (HS) membrane proteoglycans.

Our results could be summarized as follows: 1/LMWF increased endothelial cell migration and vascular tube formation; 2/LMWF modified HS- and SDC metabolism (increased heparanase (HPSE) level and activity, change in SDC-1/-4 expression and shedding); 3/EXT2, HPSE and SDC-4 are involved in LMWF cellular effects since silencing *EXT2*, *HPSE*, or *SDC-4* affect LMWF-induced angiogenesis; 4/Our data also evidenced a link between EXT2, HPSE, and SDC-4 level since silencing *EXT2* or *HPSE* led to increased translational expression of SDC-4.

Fucoidan as a heparin-like molecule can physically interact with several heparin-binding growth factors and chemokines. Fucoidan may promote or inhibit growth factor effects by trapping endogenously-released growth factors, or by displacing its ligands from their storage sites, increasing their bioavailability. Thus, LMWF has been shown to release the glycosaminoglycan-bound stromal cell-derived factor-1 (SDF-1)/CXCL12, which mobilizes progenitor cells [22]. (SDF-1)/CXCL12 may participate in angiogenesis together with vascular endothelial growth factor (VEGF) and fibroblast growth factor (FGF) [23]. In this context, one could suppose that the modification in HS chain synthesis or degradation would affect LMWF activities. For example, it has been demonstrated that the overall size of HS chains, as well as the specific features of HS chains, including the sulfated patterns, can affect FGF signaling activation [24]. Our data suggest that LMWF effects depend on HS, since LMWF-mediated angiogenesis is decreased either in *EXT2* (involved in HS biosynthesis) or *HPSE* (involved in HS degradation) silencing conditions. Furthermore, our data also demonstrated that LMWF increased HPSE expression and activity, and does not really affect EXT1 and EXT2 expression in endothelial cells. It is of note that LMWF treatment only slightly affects the HS level in our *in vitro* condition of HUVEC culture. This could be related to the law sensitivity of the dimethyl-methylene blue (DMMB) assay.

Interestingly, the inverse relations among HS, EXT1, and HPSE expressions are observed in cancer cell models. Cancer cells with higher EXT1 expression exhibited lower HPSE expression, whereas cancer cells with lower EXT1 expression exhibited higher HPSE expression [25]. In addition, the *EXT1* knockdown with siRNA led to up-regulation of HPSE expression and potentiation of metastatic capacity [25]. Similarly, Huegel *et al.* recently demonstrated that interfering with HS function, both with the chemical antagonist Surfen or treatment with bacterial heparitinase, up-regulated endogenous HPSE gene expression, suggesting a feedback mechanism that would result in further HS reduction and increased signaling [26]. With our data, it suggests that a coordinated regulation of key features of HS expression (EXT enzymes and HPSE) does exist even no mechanism has been brought out yet.

Our results also highlight a more important role of EXT2 than EXT1 in LMWF-inducted angiogenesis. This could be related to the fact that these enzymes can act differently on HS biosynthesis as demonstrated by Busse *et al.* [27].

Besides, HPSE overexpression has already been involved in *in vivo* angiogenesis in mice models. Homozygous transgenic mice that overexpress HPSE demonstrate both a deep reduction in the size of HS chains, as well as enhanced neovascularization of mammary ducts [28], some conclusions that seem consonant with our observations. The overexpression of HPSE by tumors may activate tumor angiogenesis through various mechanisms in addition to promoting the release of growth factor-decorated HS fragments. HPSE has been demonstrated to be a mediator of angiogenesis by different mechanisms [29]. HPSE promotes: 1/endothelial cell migration and degradation of the subendothelial basal lamina; 2/release of active HS-bound FGF and VEGF; 3/release of HS degradation fragments that promote FGF receptor binding, dimerization and signaling. In addition, HPSE has been demonstrated to be related to changes in the distribution of SDC-1, in particular by acting on SDC-1 ectodomain shedding [30]. We have investigated effects of LMWF on SDC-1 and SDC-4. Both heparan sulfate proteoglycans are involved in cell migration through cell cytoskeletal rearrangement, spreading, and 2D-angiogenesis. We have demonstrated that LMWF increases endothelial cell SDC-1 expression and shedding, and has an opposite effect on the same SDC-4 features. In rat injured thoracic aorta, our recent *in vivo* results demonstrate that LMWF treatment increased SDC-1 expression in the neointima layer of the injured artery, but decreased the SDC-4 expression in the neointima and media layers, therefore strengthening the *in vitro* data [10]. *SDC-4*, but not *SDC-1* silencing in HUVECs increases the LMWF-induced angiogenesis and cell migration, suggesting that SDC-4 expression partially counteracts LMWF effects.

Furthermore, our data also evidenced an unknown link between EXT2, HPSE, and SDC-4 level since silencing *EXT2* or *HPSE* led to increased translational expression of SDC-4. In these conditions, SDC-1 expression remains unchanged. These data suggested that the amount of HS present on SDC-4 core proteins could regulate the rate of SDC-4 core protein synthesis. Similarly, Ramani *et al.* recently demonstrated that HS-chains of SDC-1 regulate ectodomain shedding accompanied by a very high increase in core protein synthesis [31].

Altogether, we hypothesize that LMWF affects SDCs shedding and expression by acting through both enzymes HPSE and matrix metalloproteinase-2 [10], leading to change in the binding and the signaling and/or the bioavailability of heparin-binding proteins in the process of angiogenesis.

## 3. Experimental Section

### 3.1. Cell Culture

Human umbilical vein endothelial cells (HUVEC, N°CRL-1730, ATCC) were cultured in Endothelial Cell Basal Media 2 (PromoCell, Heidelberg, Germany) supplemented with 10% of fetal calf serum (Lonza, Levallois-Perret, France), and a mix from PromoCell containing EGF (5.0 ng/mL), Hydrocortisone (0.2 µg/mL), VEGF (0.5 ng/mL), bFGF (10 ng/mL), R3 IGF-1 (20 ng/mL), Ascorbic Acid (1 µg/mL), Heparin (22.5 µg/mL), 1% Penicillin Streptomycin (PAA Laboratories, Pasching, Austria). Cells were divided two times per week at a sub cultivation ratio of 1:3.

### 3.2. Low Molecular Weight Fucoidan

Low molecular weight fucoidan (LMWF) was isolated and hydrolyzed by a radical depolymerization process [32] from high molecular weight (HMW) extracts from *Fucus vesiculosus*, a brown seaweed (Kraeber & Co GmbH, Ellerbek, Germany). The characteristics of LMWF according to previously reported analytical methods [33] are: weight average molecular mass 8 ± 1 kDa; fucose content 35% (wt/wt); uronic acid content 3% (wt/wt); and sulfate content 34% (wt/wt). The structural model of fucoidan prepared from *Fucus vesiculosus* was determined previously by others [34,35].

### 3.3. Glycosaminoglycan Extraction

Frozen supernatant from HUVEC cell culture were freeze-dried and suspended in the extraction buffer (50 mM Tris pH 7.9, 10 mM NaCl, 2 mM MgCl_2_ and 1% of Triton X-100). Samples were digested by proteinase K (PK) (50 µg/mL final sample concentration; Merck, Molsheim, France) at 56 °C for 24 h. After PK inactivation (90 °C, 30 min), samples were treated by DNase (10 U/mL final sample concentration; Qiagen, Courtaboeuf, France) at 37 °C, overnight. Then, samples were centrifuged (13,000× *g*, 10 min) through a 0.22 µm filter unit (Pall, Saint-Germain-en-Laye, France). NaCl was added to a final concentration of 4 M and the filtered samples were vigorously agitated for 30 min. Proteins were precipitated with TCA (10% final sample concentration; Sigma-Aldrich, Saint-Quentin Fallavier, France) at 4 °C. Pellets were discarded and supernatants were cleared by chloroform washing. Finally, aqueous phases were immediately dialysis (Spectrum, 3500 MWCO) against buffer (50 mM Tris pH 7.5, 50 mM CH_3_COONa, 2 mM CaCl_2_) and then pure water before freeze-drying. Identities of the extracted GAGs were analyzed by specific digestion with chondroitinase ABC (Sigma-Aldrich, Saint-Quentin Fallavier, France), or by nitrous acid treatment as previously described [36].

### 3.4. Glycosaminoglycan Quantification

Sulfated GAGs were quantified according to the 1–9 dimethyl-methylene blue (DMMB) assay as previously described [35]. Briefly, an aliquot of each sample was pipetted and completed up to 100 µL with pure water, 1 mL of DMMB solution was added and then vigorously agitated. Then samples were centrifuged (13,000× *g*, 10 min) to sediment the GAG/DMMB complex and supernatants were discarded. The pellet was then dissolved in 250 µL of the decomplexating solution by shaking and the absorbance of the resulting solution was measured at 656 nm. A calibration curve constructed with known amounts of chondroitin sulfate (CS) A or HS standard was included in each assay.

### 3.5. Flow Cytometry Analysis

To identify the level of heparan sulfate on HUVEC cell surface, cells were preincubated for 1 h at 4 °C with anti-HS Abs (10 µg/mL, Clone 10E4; Seikagaku COGER, Paris, France) or with isotypes. After washing, cells were labeled for 30 min at 4 °C with anti-mouse Ig-FITC (1:50; Becton Dickinson, Le Pont de-Claix, France). Cells were fixed in 1% paraformaldehyde (PFA) and analyzed with a FACScan (Becton Dickinson, Le Pont de-Claix, France).

### 3.6. Real-Time RT-PCR

Real-time RT-PCR were performed using an Applied Step-One device with EXT1 (Hs00609162_m1), EXT2 (Hs00925442_m1), SDC-1 (Hs Hs00896423_m1), SDC-4 (Hs Hs01120909_m1) and Heparanase (Hs00180737_m1) TaqMan Inventoried Assay and TaqMan Gene Expression Master Mix kit (Life Technlologies, Saint Aubin, France). The mRNA levels were normalized with *GAPDH* housekeeping gene levels as described in the manufacturer’s instructions (Hs02758991_g1, TaqMan Inventoried Assay; Life Technologies, Saint Aubin, France).

### 3.7. RNA Interference

*EXT1* and *EXT2* gene-specific sense and antisense 21-nt single stranded RNAs with symmetric 2 nt 3′(2′-deoxy) thymidine overhangs validated by Life Technologies (s4891 and s4894, Silencer Select siRNA, Life Thecnologies, Saint Aubin, France). *EXT1*, *EXT*2, *SDC-1*, *SDC-4*, *Heparanase* (*HPSE*) and scramble *SNC* silencing were carried out as previously described [18,19]. HUVEC cells were transfected with 50 nM siRNA in serum-free medium using INTERFERIN transfectant reagent (Polyplus, Ozyme, Saint Quentin en Yvelines, France) following the manufacturer’s instructions. In each experiment, a negative siRNA control *SNC* (Eurogentec, Angers, France) was used as a negative control. Cells transfected with specific siRNA or *SNC*-siRNA were used 48 h post transfection for RNA analysis.

### 3.8. Migration

Cell migration was measured from 6 × 10^4^ HUVECs with Bio-coat cell migration chambers (Becton-Dickinson, Le Pont de Claix, France) [37]. Briefly, inserts of Bio-coat cell migration chamber were coated with fibronectin (100 µg/mL; Beckton Dickinson, Le Pont de-Claix, France). 6 × 10^4^ HUVECs treated with *SDC-1*-, *SDC-4*-, *EXT1*-, *EXT2*-siRNA or *SNC*-siRNA for 48 h were resuspended in basal media supplemented or not with 10 µg/mL LMWF. Cells were added in the upper chamber and complete media was added in the lower chamber. After 24 h, cells migrated through the porous membrane were stained with Mayer’s hemalum (Sigma-Aldrich, Saint-Quentin Fallavier, France) and counted manually by two different observers who performed the blind data acquisition. The cell migration rate was [(D1 − D2)/D1] × 100; D1 was the difference between the number of migrated *SNC*-siRNA-transfected cells stimulated by LMWF and that of unstimulated migrated *SNC*-siRNA-transfected cells; D2 was the difference between the number of migrated specific siRNA-transfected cells stimulated with LMWF and that of unstimulated specific siRNA-transfected cells. Alternatively, 6 × 10^4^ cells were pre-incubated or not for 2 h with the following antibodies: anti-SDC-1 (Clone DL101, IgG1; Santa Cruz Biotechnology, Heidelberg, Germany), anti-SDC-4 (Clone 5G9, IgG2a; Santa Cruz, Heidelberg, Germany) or their isotype (Becton–Dickinson, Le Pont de-Claix, France) at 5 µg/mL. The cell migration rate was [(D3 – D4)/D3] × 100; D3 was the difference between the number of migrated isotype-preincubated cells stimulated with LMWF and that of migrated unstimulated isotype-preincubated cells. D4 was the difference between the number of migrated antibodies-preincubated cells stimulated with LMWF and that of migrated unstimulated antibodies-preincubated cells.

### 3.9. Immunocytochemistry

HUVECs were harvested and put into the labtek chamber (10 × 10^4^ per well), than incubated with 10 μg/mL of LMWF for 2 h and lead to spread. Then the cells were permeabilized in 0.05% Triton X-100 (Sigma-Aldrich), stained with Alexa Fluor 546 phalloidin (F-actin, dilution 1/100; Life Technlologies, Saint Aubin, France) and lamellipodia formation were observed with a confocal microscopy (LSM 510; Carl Zeiss, Marly le Roi, France). 

### 3.10. In Vitro Angiogenesis Assay

2D-angiogenesis assay (capillary tube formation in Matrigel) was performed with 9 × 10^4^ cells/well seeded on Matrigel-coated 24-well plate (Beckton Dickinson, Le Pont de-Claix, France) and treated for 24 h with 10 µg/mL LMWF. Endothelial cells were pre-treated with *EXT1*-, *EXT2*-siRNA or *SNC*-siRNA (control) 48 h before. The capillary tubes were fixed with 4% PFA and stained with 1% Hematoxylin (Sigma-Aldrich, Saint-Quentin Fallavier, France) and photographed in phase contrast microscopy (CK40; Olympus, Rungis, France) after 24 h. The average length of vascular capillary tubes was evaluated using the open source ImageJ Software (Open Source, ImageJ ver 1.47r, National Institutes of Health, Bethesda, MD, USA).

### 3.11. Western Blot

HUVECs were incubated with 10 μg/mL of LMWF for 24 h and assayed for western blot as previously described [38]. The supernatant was collected and cell lysate protein concentration was determined by bicinchoninic acid (BCA) assay (Pierce Biotechnology, Rockford, IL, USA). Total protein was probed using anti-SDC-1 and anti-SDC-4 (respectively: mouse monoclonal IgG1, clone DL101 and rabbit polyclonal IgG, H-140, for both dilution 1/500; Santa Cruz Biotechnology, Heidelberg, Germany), anti-EXT1 and anti-EXT2 (respectively: rabbit polyclonal IgG, H-114 and goat polyclonal IgG, C-17, for both dilution 1/500; Santa Cruz Biotechnology, Heidelberg, Germany), anti-HPSE (rabbit polyclonal IgG, H-80, 1/500; Santa Cruz Biotechnology, Heidelberg, Germany), or using their isotypes (all at 1/200) and revealed with horseradish peroxidase (HRP) conjugated anti-mouse, anti-rabbit, or anti-goat IgG (dilution 1/2500; Jackson ImmunoResearch, Suffolk, UK). For comparison α- actin (rabbit polyclonal IgG, I-19-R, dilution 1/500; Santa Cruz Biotechnology, Heidelberg, Germany) was used as relevant standard house-keeping protein and revealed with horseradish peroxidase (HRP) conjugated anti-rabbit IgG (dilution 1/2500; Jackson ImmunoResearch, Suffolk, UK). Proteins were detected using Enhanced chemiluminescence detection reagents (GE Healthcare, Orsay, France). The statistical analysis was done after the protein bands quantification by Scion Image Software (Scion Corporation, Frederick, MD, USA).

### 3.12. Heparanase Activity Assay

HUVEC cell lysate or supernatant was used to determine heparanase activity using Cisbio Heparanase assay (Cisbio, Codolet, France). HepOne (InSight, Rehovot, Israel) was used for heparanase standard range. Briefly, cells lysate, supernatant, or HepOne was mixed with HS labeled with biotin and Eu3+ in reaction buffer (20 mM citrate phosphate buffer pH 5.4, 50 mM NaCl, 1 mM CaCl_2_, 0.1% BSA, 0.1% Chaps) for 3 h at 37 °C. Then for the detection step we add streptavidin-d2 (300 mM phosphate buffer pH 7, 800 mM potassium fluoride, 0.1% BSA, 2 mg/mL heparin) for 15 min at ambient temperature. Then the fluorescence was read using the following setup: excitation 337 nm, emissions 620 nm and 665 nm on M200 Pro reader (Tecan, Lyon, France).

### 3.13. Experimental Model of Intimal Hyperplasia

The experimental design was approved by the Bichat University Institutional Animal Care and use Committee (N°2011-14/698-0038). Adult male Wistar rats (*n* = 12, purchased from Janvier, CERJ, Laval, France), weighing 280 to 300 g and aged 8 weeks, were anesthetized with intraperitoneal pentobarbital (0.1 mL/kg) (CEVA Santé Animal, Libourne, France). 2F Fogerty balloon catheter (Baxter Healthcare, Maurepas, France) was inserted through an incision made in the external carotid artery and advanced along the length of the common carotid artery to the thoracic aorta [39]. The balloon was then inflated and passed three times along the length of the aortas. The balloon catheter was removed, the external carotid artery was permanently ligated and the skin wound was repaired. Then, the animals were divided into two groups: the first one received the LMWF solution (5 mg/kg/day, *n* = 6) and the second one received the saline solution (control animals, *n* = 6) via intramuscularly injection in the right leg for 14 days. Two weeks after balloon injury, rats were sacrificed by pentobarbital overdose. The thoracic aortas were harvested and divided into two groups. The first one (*n* = 6) was fixed in 4% paraformaldehyde (PFA), embedded in paraffin, and cut in 9-µm-thick cross sections for histology study. The second one (*n* = 6) was embedded in Tissue-Tek OCT Compound (Tissue-Tek, Hatfield, PA, USA), frozen in liquid nitrogen and cut in 9-µm-thick cross sections with a cryostat (Leica CM 1900, Rueil-Malmaison, France) for immunohistochemistry study.

After fixation in 4% PFA, rat aortas were stained with hematoxylin and eosin solution. Digital-slide were acquired and analyzed with a NanoZoomer (Hamamatsu, Massy, France). At least 3 sections of each stained samples were used for analysis representing different levels of the arterial segment.

Adjacent 9-µm-thick fresh arterial cross sections were immunostained with mouse anti-human endothelium CD31 (rat cross-reactive, clone RECA-1, MCA970, dilution 1/20; Abcam, Paris, France) and mouse anti-human smooth-muscle α-actin (α-SMA) (rat cross-reactive, clone 1A4, M0851, dilution 1/100; Dako, Trappes, France) as previously described [40]. Afterwards, slides were co-incubated with the appropriate secondary antibodies (5 µg/mL; Life Technologies, Saint Aubin, France). Negative control sections were incubated only with the secondary antibodies. Representative immunofluorescence photomicrographs were taken using a Leica DMRXA. Specific software (HistoLab Software, Microvision Instruments, Evry, France) allowed the tissue analysis.

### 3.14. Statistical Analysis

For the determination of statistical significance, ANOVA tests were performed with the StatView software (StatView 4.5 Abacus Concepts, Berkeley, CA, USA). All results are expressed as mean ± SEM for minimum three independent experiments (*n* = 3). A *p*-value of 0.05 was used as the criterion of statistical significance.

## Supplementary Files

Supplementary File 1
