# Supplementary material for: Heparanase and Syndecan-4 Are Involved in Low Molecular Weight Fucoidan-Induced Angiogenesis
Source: Mar Drugs. 2015 Oct 28;13(11):6588–608. doi: 10.3390/md13116588 (PMC4663543; doi:10.3390/md13116588)
Supplement: Supplementary File 1 [file marinedrugs-13-06588-s001.docx]

**Supplementary Materials**


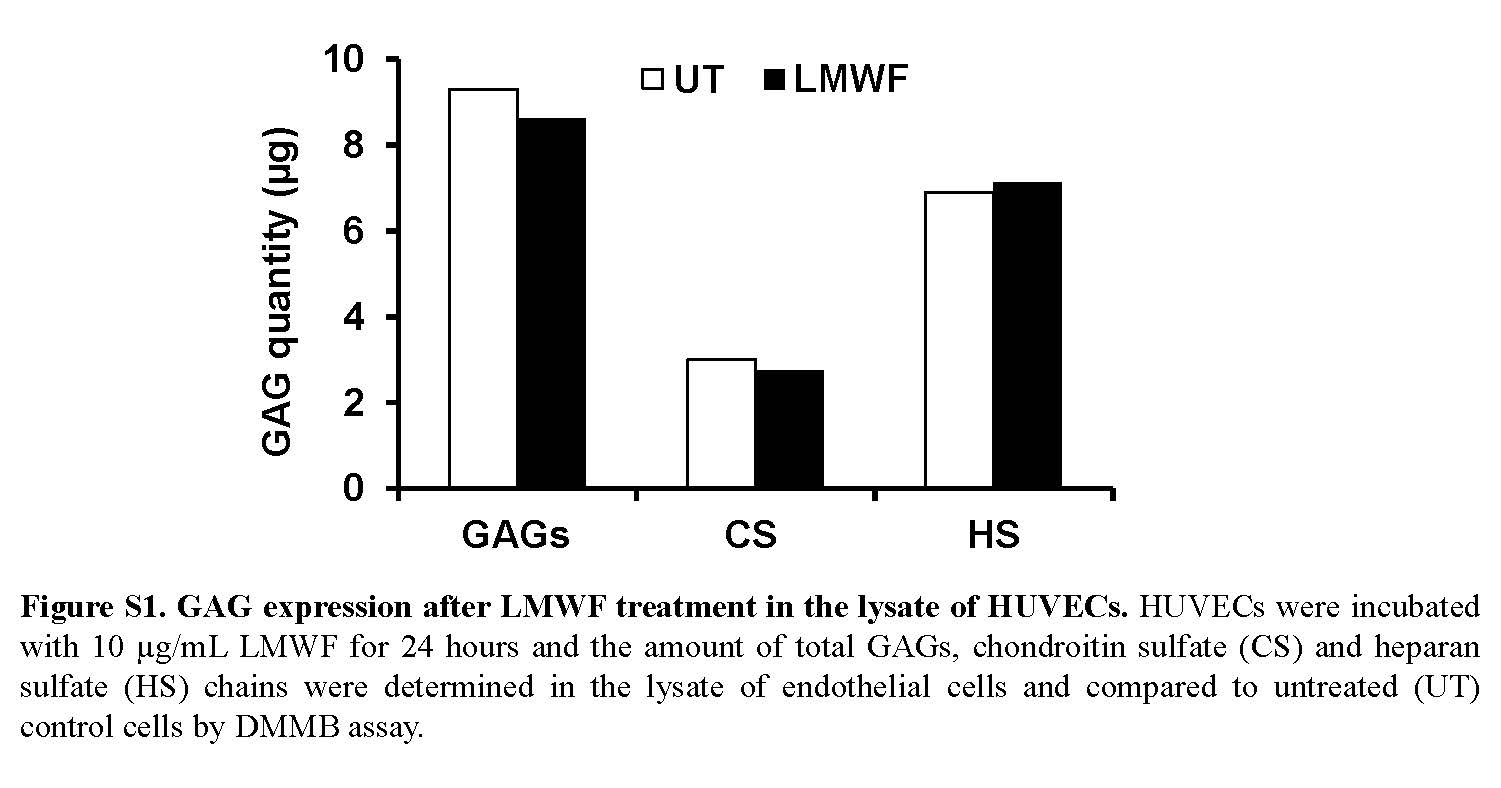


**Figure S1.** GAG expression after LMWF treatment in the lysate of HUVECs. HUVECs were incubated with 10 μg/mL LMWF for 24 h and the amount of total GAGs, chondroitin sulfate (CS) and heparin sulfate (HS) chains were determined in the lysate of endothelial cells and compared to untreated (UT) control cells by DMMB assay.


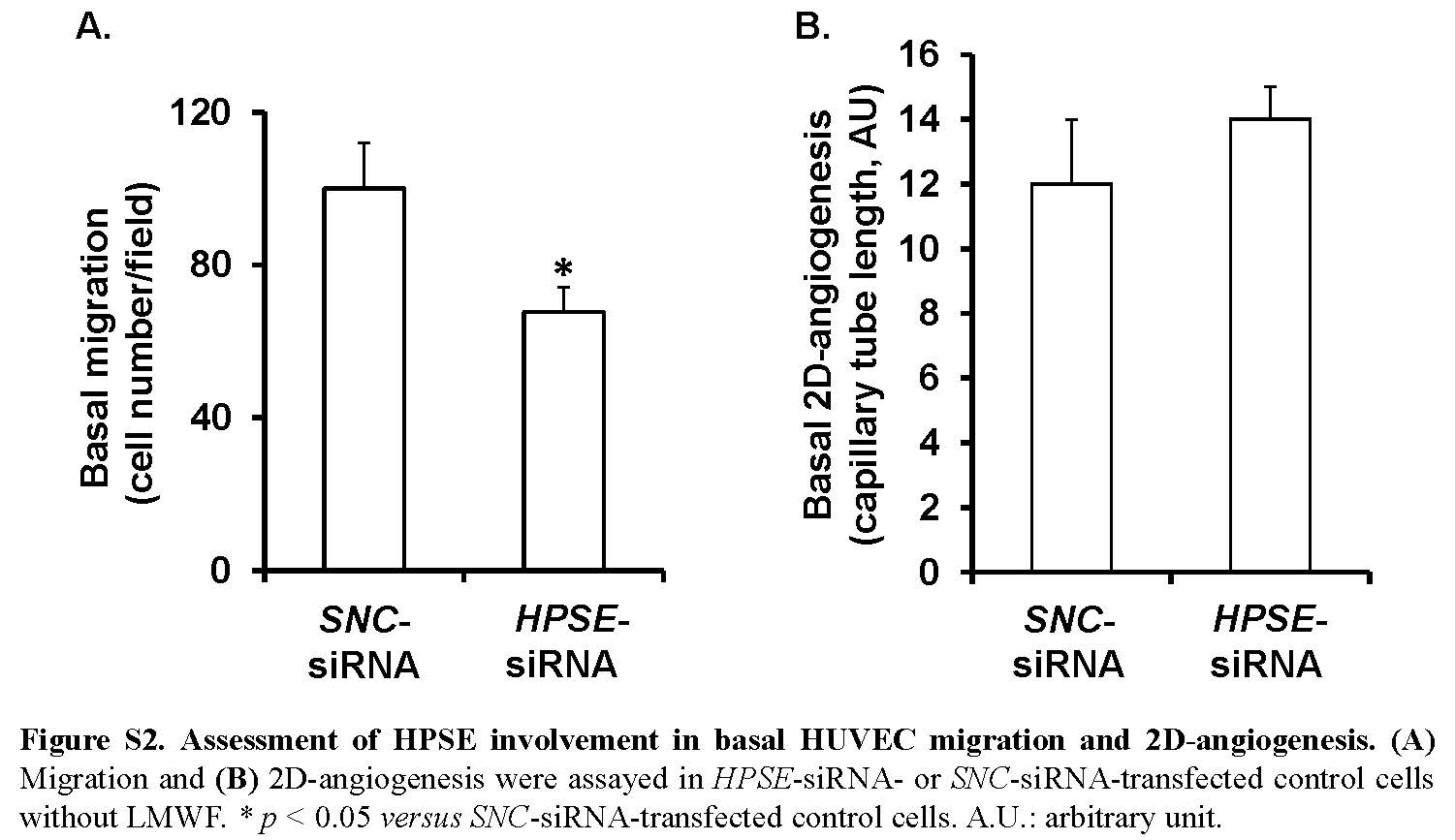


**Figure S2.** Assessment of HPSE involvement in basal HUVEC migration and 2D-angiogenesis. (**A**) Migration and (**B**) 2D-angiogenesis were assayed in *HPSE*-siRNA- or *SNC*-siRNA-transfected control cells without LMWF. ** p* < 0.05 *versus SNC*-siRNA-transfected control cells. A.U.: arbitrary unit.


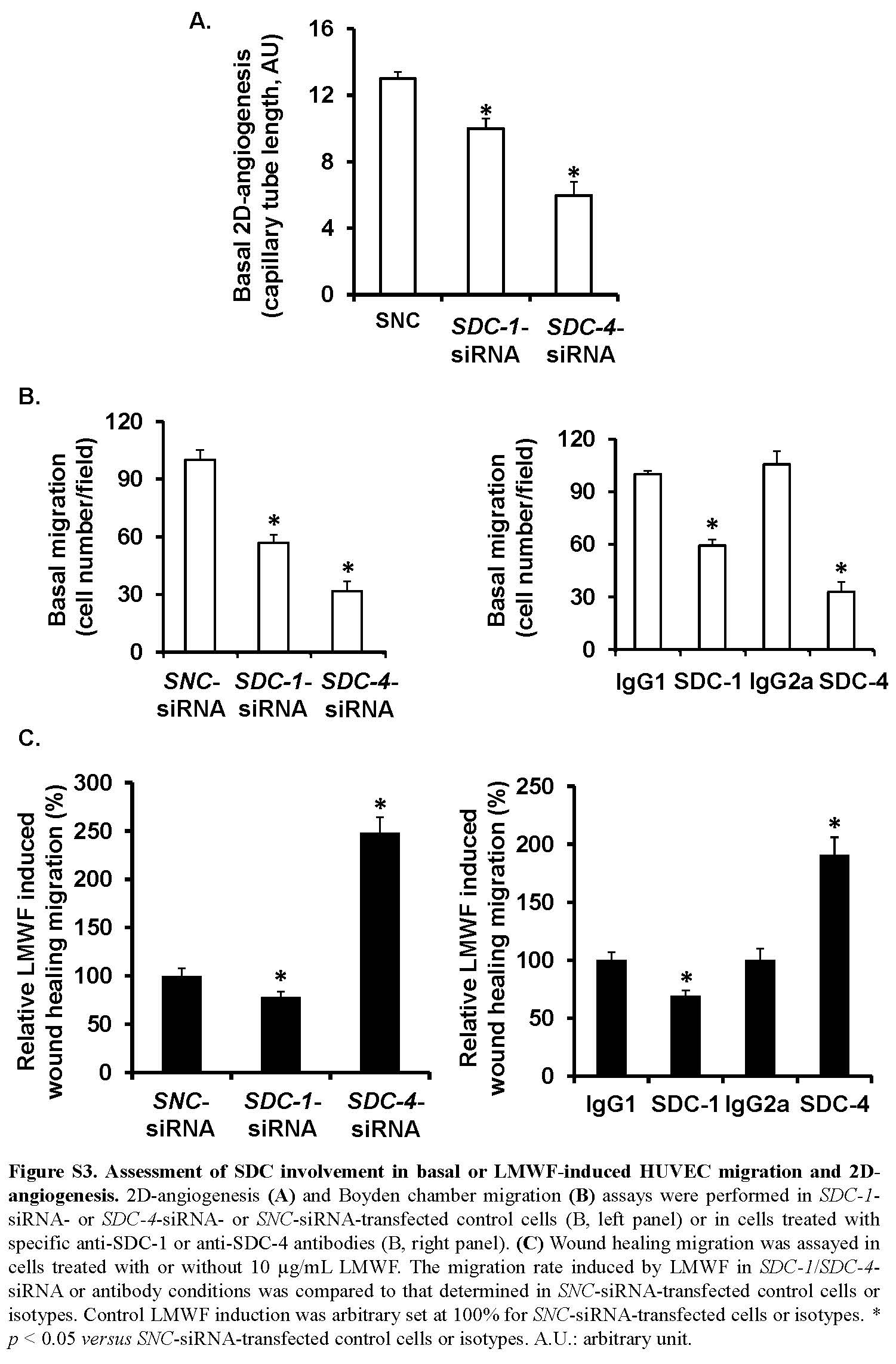


**Figure S3.** Assessment of SDC involvement in basal or LMWF-induced HUVEC migration and 2Dangiogenesis. 2D-angiogenesis (**A**) and Boyden chamber migration (**B**) assays were performed in *SDC-1*-siRNA- or *SDC-4*-siRNA- or *SNC*-siRNA-transfected control cells
(**B**, **left panel**) or in cells treated with specific anti-SDC-1 or anti-SDC-4 antibodies
(**B**, **right panel**). (**C**) Wound healing migration was assayed in cells treated with or without 10 μg/mL LMWF. The migration rate induced by LMWF in *SDC-1*/*SDC-4*-siRNA or antibody conditions was compared to that determined in *SNC*-siRNA-transfected
control cells or isotypes. Control LMWF induction was arbitrary set at 100% for
*SNC*-siRNA-transfected cells or isotypes. ** p* < 0.05 *versus SNC*-siRNA-transfected control cells or isotypes. A.U.: arbitrary unit.

© 2015 by the authors; licensee MDPI, Basel, Switzerland. This article is an open access article distributed under the terms and conditions of the Creative Commons Attribution license (http://creativecommons.org/licenses/by/4.0/).
